# A Cohort-Based Genetic Analysis of Keratoconus in Turkey Reveals a Substantial Proportion of Novel Variants and Suggests Possible Oligogenic Contributions to Keratoconus

**DOI:** 10.3390/genes17060605

**Published:** 2026-05-27

**Authors:** Barıs Paksoy, Berna Dogan, Ayşe Cengiz Ünal, Esra Kizildag Ozbay

**Affiliations:** 1 Department of Medical Genetics, Antalya Eğitim ve Araştırma Hastanesi, 07050 Antalya, Turkey; 2 Department of Ophthalmology, Antalya Eğitim ve Araştırma Hastanesi, 07050 Antalya, Turkey; bernadoga3@hotmail.com (B.D.); aysecengiz8907@gmail.com (A.C.Ü.); esrakizildag.md@gmail.com (E.K.O.)

**Keywords:** keratoconus, whole-exome sequencing, genetic heterogeneity, oligogenic inheritance, corneal ectasia, rare variants

## Abstract

**Background/Objectives:** Keratoconus is a corneal disorder that causes thinning and bulging of the cornea, resulting in astigmatism and other refractive errors. Mechanical effects and environmental factors are known to exacerbate the disease, and genetic predisposition plays a significant role in its development. **Methods:** This study investigated the presence of genetic variants in 32 keratoconus patients. We used a next-generation sequencing-based method, and variant interpretation was performed according to the American College of Medical Genetics and Genomics (ACMG) guidelines. Variants were prioritized based on multiple criteria, including population frequency data from the Genome Aggregation Database (gnomAD) (minor allele frequency < 1%), variant type and predicted functional effect, gene–disease association, inheritance pattern, phenotypic relevance, and in silico prediction tools. **Results:** Thirteen variants were identified in 11 patients (34.3%). Two patients carried variants in two different genes, raising the possibility of oligogenic contributions. Ten variants (76.9%) were novel. The variants were detected in 12 genes, namely *ADAMTS18*, *BEST1*, *CHST6*, *COL17A1*, *CYP1B1*, *KRT3*, *PAX6*, *SLC4A11*, *TACSTD2*, *UBIAD1*, *VSX1*, and *ZNF469*. No association was observed between detected variants and patient age. **Conclusions:** Our findings demonstrate a substantial proportion of novel variants and support the genetic heterogeneity of keratoconus, while also raising the possibility of oligogenic contributions in a subset of patients.

## 1. Introduction

Keratoconus is a corneal ectatic disorder that causes a decrease in corneal thickness and bulging, disrupting its proper structure and leading to reduced vision. The clinical course of keratoconus is variable; while it progresses slowly in some patients, in others, it is aggressive and begins at an early age, suggesting that the disease cannot be explained solely by mechanical and environmental factors. Recent studies have shown that cellular stress responses, oxidative damage, and disruption of the corneal stromal structure may play a role in the development of the disease. These findings support the idea that keratoconus development has a complex biological background in which genetic predisposition may play a contributory role [1]. Moreover, emerging evidence indicates that corneal homeostasis and wound healing are regulated not only by genetic variation but also by intercellular communication within the limbal niche. In particular, limbal epithelial cell-derived exosomes have been shown to transfer miRNAs and protein cargos that actively modulate limbal stromal cell proliferation, differentiation, and wound-healing responses. These findings suggest that corneal disease mechanisms may involve complex regulatory networks beyond DNA sequence variation alone, supporting a multifactorial and biologically integrated model of disease pathogenesis [2].

Keratoconus (KC) is considered a genetically heterogeneous disease with both polygenic and emerging oligogenic contributions. The variants identified in studies include not only genes related to primary corneal structural integrity and physiology but also secondary genes affecting corneal function, such as those involved in extracellular matrix organization, connective tissue pathways, and inflammatory processes. The decreasing penetrance of the identified variants and their varying frequencies across populations make variant classification difficult, leading to many variants being classified as variants of uncertain significance (VUS). All these variables explain the complex genetic basis of the disease [3].

Studies conducted in 2021 and beyond show that, in addition to isolated gene variants affecting the physiology of KC, there are genes and variants with systemic components such as collagen synthesis, cellular stress response, corneal development, and extracellular matrix organization. Next-generation sequencing data indicate that KC should be considered a multidimensional biological disorder rather than a single gene defect [4].

Although many genes associated with the disease have been repeatedly investigated in different cohorts, the number of pathogenic variants with definitively established genotype–phenotype effects remains limited. In some cases, multiple variants have been identified in different genes. This finding indicates a possible interaction between different genes in the pathogenesis. The available data indicate that the molecular genetic basis of KC is complex and may involve oligogenic contributions in some patients. Furthermore, new candidate genes and novel variants are being identified to elucidate the genetic basis of the disease [5].

In this study, we analyzed patients with a clinical diagnosis of KC in the Turkish population using whole-exome sequencing (WES). Our aim was to report rare and novel variants and to evaluate whether these findings are compatible with the genetically heterogeneous nature of the disease and possible oligogenic contributions.

## 2. Materials and Methods

### 2.1. Study Cohort and Clinical Evaluation

This study included 32 patients who underwent genetic variant analysis for corneal disease between 2022 and 2023. Patients diagnosed with keratoconus (KC) at the Ophthalmology Clinic of Antalya Training and Research Hospital were referred to the Medical Genetics Clinic for further genetic evaluation. During the study period, three patients who did not attend follow-up visits and whose clinical and topographic data were unavailable were excluded from the study.

The study was designed retrospectively and conducted in accordance with the Declaration of Helsinki. Ethical approval was obtained from the Antalya Training and Research Hospital Ethics Committee (approval number: 2023-07-06/9-32).

The diagnosis of keratoconus was based on comprehensive clinical ophthalmic examination findings and corneal topographic assessment. Each subject underwent a complete ocular assessment, including best-corrected visual acuity (BCVA) measurement, slit-lamp biomicroscopy, intraocular pressure measurement, dilated fundus examination, and corneal topography. Topographic measurements were performed using the Sirius Scheimpflug–Placido topography (Costruzione Strumenti Oftalmici, Florence, Italy). Clinical data collected for genotype–phenotype correlation included age at diagnosis, disease laterality, maximum keratometry (Kmax), corneal pachymetry and family history when available. These parameters were incorporated into the genotype–phenotype comparison analyses. For patients with bilateral involvement, corneal parameters from the more severely affected eye were included in genotype–phenotype comparisons.

All cases included in the study were sporadic, with no known familial clustering at the time of clinical evaluation. Due to the retrospective design of the study and the lack of availability of parental or additional family samples, segregation analysis could not be performed. No parental testing or extended family studies were available for any of the included patients.

### 2.2. Corneal Imaging Protocol

Corneal imaging was performed using the Sirius Scheimpflug–Placido topography system (Costruzione Strumenti Oftalmologici, Florence, Italy). This system combines a single rotating Scheimpflug camera with a Placido disk to provide a comprehensive analysis of the anterior segment.

During a single acquisition, the device captures more than 30,000 data points from the anterior and posterior corneal surfaces, along with 25 radial sections of the cornea and anterior chamber. Corneal curvature measurements were obtained in both flat and steep meridians within the central 3.0 mm zone. Corneal power and astigmatism calculations were based on a keratometric refractive index of 1.3375.

Eye movements were continuously monitored during image acquisition, and scan quality was automatically assessed by the system. In cases of poor-quality scans due to motion artifacts, misalignment, or blinking, measurements were repeated to ensure data reliability.

### 2.3. Clinical Diagnostic Criteria

Keratoconus (KC) was diagnosed based on characteristic corneal topographic and tomographic findings. Diagnostic criteria included the presence of an asymmetric bowtie pattern on corneal topography, a central keratometry value exceeding 47.2 diopters, and abnormal anterior and/or posterior corneal elevation.

**Figure 1:**
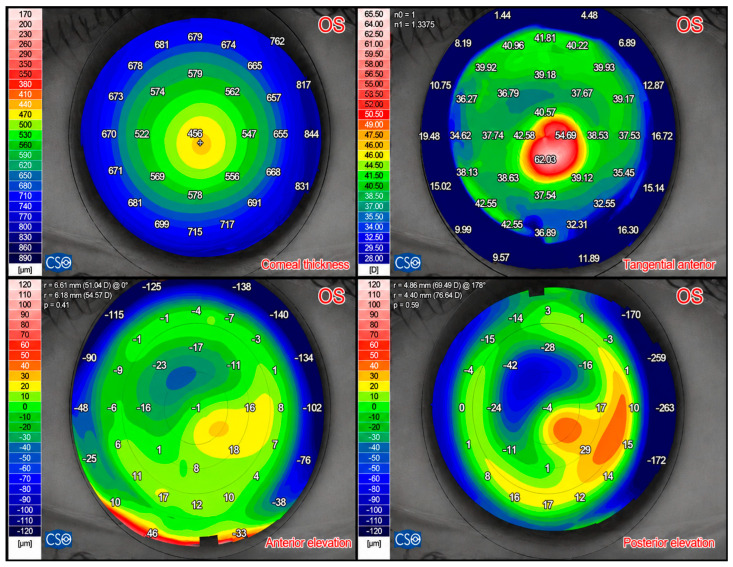
Sirius corneal topography images of the left eye of the proband harboring novel *TACSTD2* (c.55_57dup) and *VSX1*(c.619C > T) variants. The maps exhibit significant corneal thinning, asymmetric steepening, and prominent alterations in both anterior and posterior elevation. These clinical findings are highly consistent with the keratoconus phenotype.

Additional tomographic features supporting the diagnosis included localized corneal thinning and inferocentral steepening patterns. Representative corneal tomography findings consistent with keratoconus are shown in Figure 1.

### 2.4. DNA Extraction and Sequencing

Peripheral blood leukocyte DNA was extracted using the QIAamp^®^ DNA Mini Kit (Qiagen, Hilden, Germany). DNA concentration and purity were initially assessed using a NanoDrop spectrophotometer (Thermo Fisher Scientific, Wilmington, NC, USA) and subsequently quantified with the Quantus™ Fluorometer (Promega Corporation, Madison, WI, USA). Samples with an OD260/OD280 ratio between 1.8 and 2.0 were considered suitable for downstream analyses.

Whole-exome sequencing libraries were prepared using the Twist^®^ Library Preparation EF kit (Twist Bioscience, South San Francisco, CA, USA), followed by exome enrichment using the Twist^®^ exome capture system (Twist Bioscience, South San Francisco, CA, USA). Sequencing was performed on the MGI DNBSEQ-G400 (MGI Tech Co., Ltd., Shenzhen, China) platform according to the manufacturer’s instructions. Across all samples, ≥95% of target regions achieved a minimum coverage of 20×. The mean on-target coverage ranged from approximately 80× to 120×. Sequencing was conducted across multiple independent runs at different time points, reflecting routine clinical workflow conditions.

### 2.5. Bioinformatic Analysis and Variant Filtering

Raw sequencing data (FASTQ files) were uploaded to the SEQ Genomize v9.6.1 Platform (Genomize Inc., Istanbul, Turkey) for analysis. Reads were aligned to the human reference genome GRCh37 (hg19) using the Burrows–Wheeler Aligner. Variant calling was performed using FreeBayes within the platform-integrated pipeline, following standard haplotype-based variant detection algorithms. PCR duplicate removal and indel realignment were carried out using built-in algorithms of the SEQ Genomize platform.

Variant annotation was performed using Ensembl Variant Effect Predictor (VEP v102). Population frequency filtering was performed using a minor allele frequency (MAF) threshold of ≤0.01 applied globally across all populations rather than population-specific subgroups, based on gnomAD datasets integrated within the SEQ Genomize Platform and VarSome(Saphetor SA, Lausanne, Switzerland). Variant annotation and primary interpretation were conducted using the SEQ Genomize platform, while additional annotation and interpretation support were provided by VarSome, which integrates multiple genomic databases and applies ACMG-based classification criteria.

Quality filtering criteria included read depth (DP ≥ 20), genotype quality (GQ ≥ 20), and allele balance (AB ≥ 0.2 for heterozygous calls). All candidate variants were further evaluated by manual visual inspection of read alignments using the Integrative Genomics Viewer (IGV) to ensure reliability. RefSeq transcripts were used for variant annotation and reporting to maintain standardized nomenclature.

### 2.6. Variant Interpretation

Variant classification was performed in accordance with the American College of Medical Genetics and Genomics (ACMG/AMP 2015) guidelines, which were systematically and consistently applied across all identified variants to ensure standardized and reproducible variant classification. Variants were categorized as pathogenic, likely pathogenic, variants of uncertain significance (VUS), likely benign, or benign. Each applied ACMG criterion was explicitly linked to its corresponding supporting evidence to ensure transparency and reproducibility of the classification process.

Each variant was evaluated using population frequency data, predicted molecular consequence, gene–disease association, previously reported clinical evidence, and in silico pathogenicity predictions. In silico analyses were performed using multiple tools, including SIFT, PolyPhen-2, and MutationTaster, as integrated within the SEQ Genomize Platform, and were used as supporting evidence only.

Previously reported evidence was assessed using ClinVar, the Human Gene Mutation Database (HGMD®, QIAGEN Digital Insights, Redwood City, CA, USA), and Leiden Open Variation Database (LOVD), along with relevant published literature. ACMG evidence criteria applied during classification included population-based evidence (e.g., PM2), computational evidence (e.g., PP3, BP4), and previously reported disease associations. Functional validation and segregation analysis were not available for any of the identified variants in this study and therefore were not applied as evidence in variant classification.

In the absence of sufficient evidence for pathogenic or likely pathogenic classification, variants were conservatively classified as variants of uncertain significance (VUS) to avoid overinterpretation. Detailed ACMG criteria applied to each variant and their corresponding supporting evidence (including population frequency data, in silico prediction results, and ClinVar annotations where available) are provided in Table 1.

### 2.7. Statistical Analysis

All statistical analyses were performed using IBM SPSS 28.00 (IBM Corp., Armonk, NY, USA) Statistics. The distribution of continuous variables was assessed using the Shapiro–Wilk test, which indicated that the data were not normally distributed. Accordingly, non-parametric methods were employed for group comparisons. The Mann–Whitney U test was used to compare continuous variables between independent groups. Categorical variables were analyzed using the chi-square test, and when the expected cell counts were insufficient, Fisher’s exact test was applied. A two-tailed *p*-value of <0.05 was considered statistically significant.

To assess whether the identified variants were enriched compared to population-level expectations, minor allele frequencies (MAFs) were retrieved from the gnomAD genome total database. Variants with an MAF < 1% were considered rare and prioritized for analysis. Due to the limited cohort size and the heterogeneity of the detected variants across multiple genes, no formal burden or enrichment statistical tests were performed. Instead, a descriptive comparison to gnomAD genome total frequencies was conducted.

## 3. Results

### 3.1. Study Cohort and Sequencing Findings

WES was performed to analyze 32 individuals who had been clinically diagnosed with KC. Eleven patients (34.3%) had potentially disease-associated variants, while twenty one patients (65.7%) had no reportable variants (Table 2). A possible multi-variant pattern involving different genes was observed in the two patients who had two distinct variants in two different genes, all of which were novel (Table 3).

### 3.2. Distribution of Variants and Novel Variant Rate

In our KC cohort, 10 of 13 detected variants (76.9%) were novel, whereas three variants had been previously reported in genetic databases. Variants were distributed across 12 different genes, with *COL17A1* harboring more than one variant. The detected variants involved the *ADAMTS18*, *BEST1*, *CHST6*, *COL17A1*, *CYP1B1*, *KRT3*, *PAX6*, *SLC4A11*, *TACSTD2*, *UBIAD1*, *VSX1*, and *ZNF469* genes. Patients carrying more than one variant had alterations located in different genes, supporting genetic heterogeneity and raising the possibility of oligogenic contributions.

### 3.3. Age Distribution

When age distribution was evaluated according to mutation status, the mean age was 28.45 ± 10.43 years in the variant-positive group and 27.76 ± 10.41 years in the variant-negative group. Median (IQR) values were 27.00 (18.25) and 24.00 (11.00), respectively. No statistically significant difference was observed between the groups (*p* = 0.94). These findings suggest that age distribution was independent of mutation status (Table 4).

### 3.4. Comparison of Corneal Topographic and Pachymetric Parameters by Variant Status

Corneal topographic and pachymetric parameters according to mutation status are summarized in Table 4. Group comparisons were performed using the Mann–Whitney U test, and no statistically significant differences were observed for any evaluated parameter (all *p* > 0.05).

Maximum keratometry (Kmax), representing the steepest corneal curvature, showed comparable values between the mutation-positive and mutation-negative groups (*p* = 0.70). Similarly, flat keratometry (K1), reflecting the flat corneal meridian, and steep keratometry (K2), representing the steep corneal meridian, did not differ significantly between the groups (*p* = 0.48 and *p* = 0.68, respectively).

Pachymetric measurements also demonstrated similar distributions regardless of mutation status. The thinnest corneal thickness (TCT), indicating the minimum corneal thickness, showed no statistically significant difference between the groups (*p* = 0.99). Likewise, central corneal thickness (CCT), representing corneal thickness at the central apex, was comparable in mutation-positive and mutation-negative patients (*p* = 0.67).

Overall, these findings suggest that mutation status was not associated with significant differences in corneal curvature or corneal thickness parameters within this cohort.

### 3.5. Comparison of Categorical Clinical and Topographic Features According to Mutation Status

Categorical clinical and topographic features according to mutation status are summarized in Table 5. Group comparisons were performed using the chi-square or Fisher’s exact test, and no statistically significant differences were identified between mutation-positive and mutation-negative patients for any evaluated parameter (*all p* > 0.05).

Eye laterality distribution was comparable between the groups (*p* = 0.99). Similarly, cone localization analysis demonstrated no statistically significant differences between mutation-positive and mutation-negative patients for central or paracentral cone involvement (*p* = 0.99).

In addition, the distribution of Amsler–Krumeich stages did not differ significantly between the groups (*p* = 0.08), indicating comparable disease severity profiles between mutation-positive and mutation-negative patients in this cohort.

Overall, these findings suggest that mutation status was not significantly associated with categorical clinical characteristics, cone localization, or keratoconus stage in this cohort.

## 4. Discussion

With the increasing number of genetic studies on KC, it has become clear that the disease is not merely a structural defect of the cornea. Instead, genes involved in corneal structure and inflammatory responses associated with oxidative stress and environmental factors, as well as genes involved in extracellular matrix organization, appear to contribute to disease pathogenesis. The genetic alterations identified in our study were not restricted to a single gene or biological pathway; rather, they involved several genes involved in ocular and corneal biology. In this context, our investigation is the first cohort-based genetic study of KC in Turkey and provides additional evidence for the genetic contribution to the disease in the existing literature. Taken together, our findings support the concept that keratoconus is genetically heterogeneous. In a small subset of patients, the presence of variants in more than one gene may be compatible with possible oligogenic contributions; however, these findings should be interpreted cautiously, given the limited cohort size and the absence of segregation or functional analyses.

KC genetics has been described as having a polygenic architecture based on findings from genome-wide association studies (GWAS). A multi-ethnic GWAS study [6] analyzing 4669 cases and 116,547 controls found significant associations at 36 loci, particularly highlighting the potential pathogenetic role of genes associated with corneal collagen matrix components (*COL12A1*, *COL6A1*, *COL1A1*, *COL5A1*) and genes involved in cellular differentiation/homeostasis (*KLF5*, *SMAD3*-related regions). The study emphasizes the importance of rare genetic variants. In another study, a Chinese cohort including 157 KC probands and 445 first-degree relatives revealed, through Penrose, simple, and complex segregation analyses, that KC cannot be explained by Mendelian inheritance models and exhibits a multifactorial inheritance pattern [7]. In our study, variants were distributed across multiple genes and biological pathways, consistent with the complex and multifactorial nature of keratoconus.

In the present cohort, several variants were identified in genes classically associated with autosomal recessive inheritance, including *CYP1B1*, *CHST6*, *SLC4A11*, and *TACSTD2*, but these variants were detected in the heterozygous state. Therefore, these findings were not interpreted as definitive monogenic causes of keratoconus. Instead, they were considered candidate contributory variants that may potentially act within a broader framework of genetic susceptibility. Previous studies by Pasutto et al. and Reis et al. demonstrated that heterozygous *CYP1B1* variants may contribute to ocular disease phenotypes as susceptibility or modifier factors rather than direct Mendelian causes, particularly in genetically complex disorders involving anterior segment abnormalities and glaucoma-related phenotypes [8,9]. In addition, González-Atienza et al. highlighted substantial locus heterogeneity and the presence of rare variants distributed across multiple genes and biological pathways in keratoconus, supporting the possibility that oligogenic mechanisms may contribute to disease susceptibility in a subset of patients [10]. However, in the absence of segregation analysis and functional validation, such variants should be interpreted cautiously and should not be considered sufficient evidence for direct pathogenic causality.

Recent family-based studies are revealing novel gene candidates in the pathogenesis of KC. In a study conducted in China, Lin et al. [11] identified novel variants in the candidate genes *HOMER3*, *IGF1R*, *EML6*, *DOP1B*, and *NBEAL2*, most of which were classified as variants of uncertain significance (VUS), highlighting the difficulty of interpreting novel variants without functional validation. In a study by Xu, L. and colleagues [12], 32 rare variants were identified in 13 genes in a cohort of 5 Chinese families, and it was reported that *EPCAM*, *SHROOM3*, *SYNE1*, *TEK*, and *TTN* genes were suggested as potential candidate genes involved in cell communication pathways and extracellular matrix organization. Similarly, in a study focusing solely on the *ZNF469* gene in a cohort of 25 KC patients in the Han Chinese population, five novel variants and two cases of compound heterozygosity were identified [13]. In a study conducted by WY Cheng et al. in five Chinese families, a total of six novel variants were reported in the *HMX1*, *SLC4A11*, *TGFBI*, *PIKFYVE*, and *ZEB1* genes, and segregation analysis was performed. This study identified different inheritance patterns and presented findings supporting genetic heterogeneity [14]. In all four studies in the literature, the limited sample size and lack of functional studies limit pathogenic interpretation. In contrast, our study found that novel variants in a sporadic KC cohort were distributed along different biological pathways. Therefore, data obtained from different populations are important for a more complete understanding of the global genetic architecture of KC. In this respect, our cohort represents a different population background and adds further evidence to the expanding genetic spectrum of the disease.

WES research on non-Asian ethnic groups provided important insights into the genetic architecture of KC. In a study of 24 families in a Spanish cohort, González-Atienza et al. identified numerous novel variations in the corneal and extracellular matrix structures [10]. This suggests that the biology of KC exhibits locus heterogeneity, in which various genes contribute to similar clinical manifestations. This consistency across populations supports the argument that KC is a complex genetic condition rather than an ordinary Mendelian inheritance pattern.

Using targeted next-generation sequencing, Lombardo and his team recently examined the genetic landscape of KC in an Italian cohort [15]. They created a custom panel of 26 candidate genes and tested it on 64 patients. They found 167 different alleles across 22 genes. *FLG*, *ZNF469*, *DOCK9*, and *LOXHD1* had the most variants in their sample. They also found that some variants were more common in people with progressive disease than in people with stable KC, which is interesting. Instead of identifying a single dominant gene, we found variants spread out over several loci. These findings are compatible with the possibility that oligogenic mechanisms may contribute to disease susceptibility in a subset of KC patients [16].

Selected single-nucleotide polymorphisms (SNP typing) in genes such as *FOXO1* and *ZNF469*, which are associated with genetics, have been examined. In contrast, next-generation sequencing (NGS) allows for the investigation of numerous genes and all their variants. In cohorts with small sample sizes, as in the literature and our study, NGS increases the probability of variant detection. Studies conducted with NGS provide more comprehensive information about the genetic structure of the disease compared to SNP typing. The fact that 13 variants were detected in 32 patients in our study shows that the variants are not concentrated in a single gene but that changes were detected in different genes in different patients. The difference between these two studies mainly stems from the methods used. NGS-based cohort studies offer an expanded perspective of the genetic basis of the disease, while candidate gene or SNP-based association studies are essential for confirming previously reported genetic risk regions. Consequently, evaluating different methods collectively improves our understanding of the genetic basis for KC [15].

Previous studies on the genetics of KC in Turkey have largely focused on single genes or specific polymorphisms. While limited studies focusing on the *VSX1* and *ZNF469* genes [17,18] SNP association analyses performed on genes such as *IL1B*, *IL1RN*, and *GPX1* genes [19,20] have contributed to the evaluation of the disease within the framework of specific candidate genes, the limited gene coverage has not fully revealed genetic heterogeneity. However, the lack of significant variant detection in miRNA-based variant screenings [21] suggests that KC cannot be explained by a single gene or a single biological pathway. It is noteworthy that most of these studies kept the number of genes limited and did not explore potential multi-gene contributions. In contrast, our study, using a WES approach, identified variants across multiple genes and revealed a high proportion of novel variants. This highlights the added value of comprehensive sequencing compared to candidate gene studies.

Recent evidence further supports the role of miRNA dysregulation and oxidative stress pathways in corneal disease biology. Zha et al. demonstrated that miR-10b-5p may contribute to oxidative stress-related corneal dysfunction through suppression of *GCLM* and *LANCL1*, leading to impaired antioxidant defense mechanisms and disruption of redox homeostasis in diabetic corneal tissue [22]. Their integrated multi-omics analysis additionally highlighted the potential contribution of miRNA-mediated regulatory pathways to corneal epithelial dysfunction and wound-healing abnormalities. These findings support the concept that corneal diseases may involve complex molecular and regulatory mechanisms extending beyond structural gene variation alone.

A total of 13 unique variants were identified; 12 were classified as variants of uncertain significance (VUS) and one as likely pathogenic according to ACMG criteria. In this study, WES was applied to all cases, and the identified variants were classified according to ACMG criteria. The findings suggest that rare and novel variants may be present in Turkish patients with keratoconus. However, since the majority of variants were classified as variants of uncertain significance (VUS), these results should not be interpreted as direct evidence of causality but rather as candidate genetic findings that may contribute to disease biology. Although the detection of multiple variants in some patients may suggest a possible oligogenic contribution, this interpretation requires further validation through segregation analyses, functional studies, and studies with larger cohorts. It is thought that this finding is related to the elevated frequency of novel variants in both studies. Because novel variants have not been clinically documented before, and the genotype–phenotype relationship is not well known, these variants are typically classified as VUS. Further studies are needed to clarify the pathogenicity of these variants. Moreover, the lack of next-generation sequencing research on KC genetics highlights the necessity for more extensive studies in this field [16].

## 5. Conclusions

In conclusion, our cohort-based study in the Turkish population provided data regarding corneal pathologies with a broad distribution of variants across 12 genes and high rates of novel variants, providing additional evidence supporting the genetic contribution to keratoconus. The detection of variants in different genes in some patients may indicate possible oligogenic contributions; however, further functional and segregation studies are required to clarify their biological relevance. Overall, our findings support the genetically heterogeneous nature of KC and are compatible with the possibility that oligogenic contributions may be present in a subset of patients.

## Figures and Tables

**Table 1:** Detailed ACMG classification of identified variants.

Variant (HGVS)	Genetic Context	GnomAD AF	ClinVar	ACMG Evidence	Class
*CYP1B1* c.182G > A p.Gly61Glu	AR (Het), missense Reported	0.000217	Pathogenic	PP5; PM2; PP3	VUS
*ADAMTS18* c.3157C > T p.Arg1053Trp	AR(Het) Missense reported	0.00128	Conflicting (B/VUS)	PM2; PP3	VUS
*COL17A1* c.134C > T p.Ser45Phe	AR/AD (Het)/Missense Novel	NA	Not Reported	PM2; PP3	VUS
*PAX6* c.565 + 1G > A p.?	AD (Het), splice donor, novel	NA	Not Reported	PVS1; PM2	LP
*CHST6* c.614G > A p.Arg205Gln	AR (Het), missense reported	0.0000131	Not Reported	PM2; PP3	VUS
*BEST1* c.856G > A p.Gly286Ser	AD (Het), missense, novel	NA	Not Reported	PM2; PP2; PP3	VUS
*UBIAD1* c.134A > C p.Lys45Thr	AD (Het), missense, novel	NA	Not Reported	PM2; PP3	VUS
*KRT3* c.325_336del p.Phe109_Gly112del	AD (Het), in-frame deletion, novel	0.000496	Not Reported	PM2; PM4	VUS
*ZNF469* c.11537G > A p.Arg3846Gln	AR (Het), missense, novel	NA	Not Reported	PM2	VUS
*COL17A1* † c.2230C > T p.Pro744Ser	AR/AD (Het), missense/splice region, novel	NA	Not Reported	PM2; PP3	VUS
*SLC4A11* † c.1895C > T p.Ala632Val	AR (Het), missense, novel	0.00000657	Not Reported	PM2; PP3	VUS
*TACSTD2* ‡ c.55_57dup p.Leu19dup	AR (Het), in-frame duplication, novel	0.000192	Not Reported	PM2; PM4	VUS
*VSX1* ‡ c.619C > T p.Arg207Trp	AD (Het), missense, novel	0.0000638	Not Reported	PM2; PP3	VUS

Abbreviations: VUS, variant of uncertain significance; LP, likely pathogenic; Het, heterozygous; NA, not available; B, benign; T, tolerated; AD, autosomal dominant; AF, allele frequency; AR, autosomal recessive. Population frequencies were obtained from the gnomAD Exomes and Genomes datasets. Variant classification was performed according to the American College of Medical Genetics and Genomics (ACMG) guidelines. In silico predictions were derived from multiple algorithms (SIFT, PolyPhen-2, MutationTaster) integrated within the SEQ Platform and VarSome. ClinVar annotations were used as supportive evidence when available. † Variants identified in the same patient. ‡ Variants identified in another patient with multiple variants.

**Table 2:** Summary of genetic findings in the study cohort.

Parameter	Value
Total patients (*n*)	32
Variant-positive patients, *n* (%)	11 (34.3%)
Total variants detected, *n*	13
Novel variants, *n* (%)	10 (76.9%)
Previously reported variants, *n* (%)	3 (23.1%)
Genes with detected variants, *n*	12

Abbreviations: *n*, number of cases; %, percentage of the total variants or patients.

**Table 3:** Genotype–phenotype characteristics of keratoconus patients with identified variants.

Variant (HGVS)	Genetic Context	Clinical Findings (Kmax/TCT)	ClinVar	Class
*CYP1B1* c.182G > A (Reported) p.Gly61Glu	AR (Het) *Missense*	50.12/443	P	VUS
*ADAMTS18* c.3157C > T (Reported) p.Arg1053Trp	AR (Het) *Missense*	130.87/320	B/VUS	VUS
*COL17A1* c.134C > T (Novel) p.Ser45Phe	AR/AD (Het) *Missense*	65.26/491	NR	VUS
*PAX6* c.565 + 1G > A (Novel) p.?	AD (Het) *Splice Donor*	58.8/385	NR	LP
*CHST6* c.614G > A (Reported) p.Arg205Gln	AR (Het) *Missense*	50.31/470	NR	VUS
*BEST1* c.856G > A (Novel) p.Gly286Ser	AD (Het) *Missense*	54.7/511	NR	VUS
*UBIAD1* c.134A > C (Novel) p.Lys45Thr	AD (Het) *Missense*	58.72/422	NR	VUS
*KRT3* c.325_336del (Novel) p.Phe109_Gly112del	AD (Het) *In-frame* *Deletion*	61.23/411	NR	VUS
*ZNF469* c.11537G > A (Novel) p.Arg3846Gln	AR (Het) *Missense*	61.1/347	NR	VUS
*COL17A1* † c.2230C > T (Novel) p.Pro744Ser	AR/AD (Het) *Missense*, *Splice Region*	54.94/487	NR	VUS
*SLC4A11* † c.1895C > T (Novel) p.Ala632Val	AR (Het) *Missense*	54.94/487	NR	VUS
*TACSTD2* ‡ c.55_57dup (Novel) p.Leu19dup	AR (Het) *In-frame* *Insertion*	62.03/456	NR	VUS
*VSX1* ‡ c.619C > T (Novel) p.Arg207Trp	AD (Het) *Missense*	62.03/456	NR	VUS

Abbreviations: AD, autosomal dominant; AR, autosomal recessive; Het, heterozygous; HGVS, Human Genome Variation Society nomenclature; Kmax, maximum keratometry; LP, likely pathogenic; NA, not available; NR, not reported; P, pathogenic; TCT, thinnest corneal thickness; VUS, variant of uncertain significance. Variants marked with † were identified in the same patient, whereas variants marked with ‡ were identified in another patient carrying multiple variants.Topographic patterns consistent with keratoconus were observed in all variant-positive cases.

**Table 4:** Comparison of ocular measurements according to variant status.

Variable	Mutation-Positive		Mutation-Negative		*p*
	X ± s.s.	IQR	X ± s.s.	IQR	
Age (years)	28.45 ± 10.43	27.00 (18.25)	27.76 ± 10.41	24.00 (11.00)	0.94
Kmax	58.20 ± 17.23	54.62 (10.22)	55.57 ± 9.53	54.82 (8.65)	0.70
K1	45.94 ± 4.30	44.81 (5.40)	45.51 ± 5.29	44.80 (4.45)	0.48
K2	49.12 ± 6.00	46.83 (6.99)	48.64 ± 5.92	46.88 (5.83)	0.68
TCT	447.09 ± 55.92	449.50 (67.25)	441.57 ± 57.99	458.00 (79.75)	0.99
CCT	469.81 ± 45.67	472.00 (56.00)	457.88 ± 54.60	467.00 (75.00)	0.67

Abbreviations: Kmax, maximum keratometry; K1, flat keratometry; K2, steep keratometry; TCT, thinnest corneal thickness; CCT, central corneal thickness; IQR, interquartile range.

**Table 5:** Comparison of clinical and topographic characteristics according to mutation status.

Variable	Category	Mutation-Positive *n* (%)	Mutation-Negative *n* (%)	*p*
Eye Laterality	Bilateral	10 (90.9)	19 (90.5)	0.99
	Unilateral	1 (9.1)	2 (9.5)	
Cone Location	Central	1 (9.1)	2 (9.5)	0.99
	Paracentral	10 (90.9)	19 (90.5)	
Amsler–Krumeich Stage	Stage 1	0 (0.0)	2 (9.5)	0.08
	Stage 2	2 (18.2)	3 (14.3)	
	Stage 3	2 (18.2)	11 (52.4)	
	Stage 4	7 (63.6)	5 (23.8)	

## Data Availability

The genetic and clinical data generated in this study are not publicly available due to patient confidentiality, ethical restrictions, and local data protection regulations. The study was conducted using retrospective anonymized clinical and genetic data, and unrestricted public sharing of individual-level genomic data is limited under institutional ethical policies and the Turkish Personal Data Protection Law (KVKK, Law No. 6698), particularly Article 6 regulating the processing of sensitive personal data, including genetic and health-related information. De-identified data may be made available from the corresponding author upon reasonable request and subject to institutional ethical approval, where applicable.

## Abbreviations

The following abbreviations are used in this manuscript:

ACMG	American College of Medical Genetics and Genomics
BCVA	Best-corrected visual acuity
IQR	Interquartile range
KC	Keratoconus
MAF	Minor allele frequency
NGS	Next-generation sequencing
VUS	Variant of uncertain significance
WES	Whole-exome sequencing
